# Severity Scale of Influenza and Acute Respiratory Illness Hospitalizations to Support Viral Genomic Surveillance: A Global Influenza Hospital Surveillance Network Pilot Study

**DOI:** 10.1111/irv.70085

**Published:** 2025-03-06

**Authors:** Bronke Boudewijns, Saverio Caini, Marco Del Riccio, Marta C. Nunes, Sandra S. Chaves, Melissa K. Andrew, Justin R. Ortiz, Oana Săndulescu, Joseph S. Bresee, Elena Burtseva, Daouda Coulibaly, Daria M. Danilenko, Kirill Stolyarov, Anca C. Drăgănescu, Mine Durusu Tanriover, Heloisa I. G. Giamberardino, Parvaiz A. Koul, F. Xavier Lopez‐Labrador, Shelly A. McNeil, Ainara Mira‐Iglesias, Alejandro Orrico‐Sanchez, Nancy A. Otieno, Jorim Ayugi, Sonia M. Raboni, Peter Spreeuwenberg

**Affiliations:** ^1^ Netherlands Institute for Health Services Research (Nivel) Utrecht the Netherlands; ^2^ Department of Health Sciences University of Florence Florence Italy; ^3^ Center of Excellence in Respiratory Pathogens (CERP), Hospices Civils de Lyon (HCL) and Centre International de Recherche en Infectiologie (CIRI), Équipe Santé Publique, Épidémiologie et Écologie Évolutive des Maladies Infectieuses (PHE3ID), Inserm U1111, CNRS UMR5308, ENS de Lyon Université Claude Bernard Lyon 1 Lyon France; ^4^ South African Medical Research Council, Vaccines and Infectious Diseases Analytics Research Unit, Faculty of Health Sciences University of the Witwatersrand Johannesburg South Africa; ^5^ Foundation for Influenza Epidemiology, Fondation de France Paris France; ^6^ Department of Medicine Dalhousie University Halifax Canada; ^7^ Canadian Center for Vaccinology Dalhousie University Halifax Canada; ^8^ Center for Vaccine Development and Global Health University of Maryland School of Medicine Baltimore Maryland USA; ^9^ Department of Infectious Diseases I, Faculty of Medicine Carol Davila University of Medicine and Pharmacy Bucharest Romania; ^10^ National Institute for Infectious Diseases "Prof.Dr. Matei Balș" Bucharest Romania; ^11^ Task Force for Global Health Decatur Georgia USA; ^12^ Gamaleya Federal Research Center for Epidemiology and Microbiology Ministry of Health of Russian Federation Moscow Russia; ^13^ Epidémiologie ‐ Santé Publique Institut National d'Hygiène Publique (INHP) Abidjan Côte d’Ivoire; ^14^ Smorodintsev Research Institute of Influenza St. Petersburg Russia; ^15^ Department of Pediatrics, Faculty of Medicine Carol Davila University of Medicine and Pharmacy Bucharest Romania; ^16^ Hacettepe University Vaccine Institute Ankara Türkiye; ^17^ Turkish Society of Internal Medicine Ankara Türkiye; ^18^ Epidemiology and Infection Control/Immunization Department Pequeno Principe Hospital Curitiba Brazil; ^19^ Sheri Kashmir Institute of Medical Sciences Srinagar India; ^20^ Virology Laboratory, FISABIO‐Public Health, Generalitat Valenciana Valencia Spain; ^21^ Department of Microbiology and Ecology University of Valencia Medical School Valencia Spain; ^22^ CIBERESP Network Center for Epidemiology and Public Health Instituto de Salud Carlos III Madrid Spain; ^23^ Vaccine Research Department Fisabio‐Public Health Valencia Spain; ^24^ Universidad Católica de Valencia San Vicente Mártir Valencia Spain; ^25^ Kenya Medical Research Institute (KEMRI) Nairobi Kenya; ^26^ Molecular Biology/Microbiology Research Laboratory Universidade Federal Do Paraná Curitiba Brazil

**Keywords:** influenza, acute respiratory infection, severity

## Abstract

**Background:**

This study aimed to establish a Severity Scale for influenza and other acute respiratory infections (ARI), requiring hospitalization, for surveillance and research purposes (the SevScale). Such a scale could aid the interpretation of data gathered from disparate settings. This could facilitate pooled analyses linking viral genetic sequencing data to clinical severity, bringing insights to inform influenza surveillance and the vaccine strain selection process.

**Methods:**

We used a subset of data from the Global Influenza Hospital Surveillance Network database, including data from different geographical areas and income levels. To quantify the underlying concept of severity, an item response model was developed using 16 indicators of severity related to the hospital stay. Each patient in the dataset was assigned a Severity Score and a Severity Category (low, medium, or high severity). Finally, we compared the model scores across different subgroups.

**Results:**

Data from 9 countries were included, covering between 4 and 11 seasons from 2012 to 2022, with a total of 96,190 ARI hospitalizations. Not for all severity indicators data were available for all included seasons. Subgroups with a high percentage of patients in the high Severity Category included influenza A(H1N1)pdm09, age ≥ 50, lower‐middle income countries, and admission since the start of the COVID‐19 pandemic.

**Conclusions:**

The initial model successfully highlighted severity disparities across patient subgroups. Repeating this exercise with new, more complete data would allow recalibration and validation of the current model. The SevScale proved to be a promising method to define severity for influenza vaccine strain selection, surveillance, and research.

## 1 Introduction

Acute respiratory infections (ARIs) and in particular influenza embody a significant burden for healthcare systems worldwide. In the years before the SARS‐CoV‐2 pandemic, seasonal influenza was estimated to cause 3–5 million cases of severe illness and about 290,000–650,000 respiratory deaths each year, globally [1, 2, 3]. The Global Burden of Disease study estimated that influenza caused 9.5 million hospitalizations with lower respiratory tract infection in 2017 (range: 3.7–23 million) [4]. Influenza vaccine effectiveness (IVE) varies over time, depending on, among other factors, which strains are circulating and how well they match with that included in the vaccine. Nevertheless, vaccination substantially decreases the number of influenza‐related infections, hospitalizations, and deaths [5, 6, 7]. The Global Initiative on Sharing All Influenza Data (GISAID) plays an important role in informing the vaccine selection procedure led by the WHO Global Influenza Surveillance and Response System (GISRS) by facilitating the sharing of influenza virus genetic sequencing data [8, 9].

One of the organizations providing data to the GISAID database is the Global Influenza Hospital Surveillance Network (GIHSN), which beside its main focus on influenza, collects data on other viral ARIs as well [10]. Since its inception in 2012, the objectives of the GIHSN have evolved. Initially focused on IVE evaluation and burden of influenza disease estimates, the network's focus shifted in 2019 to encompass generation of epidemiological and medical evidence on hospitalized patients with respiratory viruses beyond influenza and to support WHO influenza vaccine strain selection procedures. As such, sites contribute whole genome sequencing data from specimens collected from hospitalized patients with laboratory‐confirmed influenza. These results are uploaded to GISAID and integrated into the clinical and demographic information collected as part of the GIHSN.

The GIHSN sequencing data represent hospitalized patients. However, comprehending the extent of disease severity across different age groups and geographic settings is challenging, due to differences in resources, local guidelines and access to care. Moreover, surveillance systems usually collect limited clinical information. The recent paper by Lina et al. [11] noted that there “is currently no consensus on how to define complicated hospitalization for influenza.” Rath et al. developed a scale for influenza severity in children [12]. However, no comparable scale is available for adult patients, and this type of composite score needs available data on all of the score's indicators of severity. Establishing a reliable Severity Scale (SevScale) for patients with ARI, and in particular influenza, could facilitate linking the score with viral genetic sequencing data. This linkage may help identify specific genetic evolution markers or mutations associated with disease severity or vaccine immune escape, thereby aiding in the selection of appropriate vaccine strains. Moreover, epidemiological studies utilizing the SevScale score as an outcome variable would achieve greater comparability, given the diversity within the GIHSN, which encompasses over 20 countries with varying geography and economies. Evidence suggests clustering of severe hospitalized patients within specific influenza subtypes or lineages, based on ICU admissions, mechanical ventilation usage, and in‐hospital deaths [13]. However, there is currently no standardized system in place to assess clinical severity within the GIHSN or other multisite surveillance platform. Moreover, now that global surveillance efforts are moving towards more integrated surveillance of respiratory viruses, it is appropriate to take a step further and develop a broad SevScale that can be implemented for ARI patients associated with a wider range of viral aetiologies than influenza. We aimed to develop a scientifically based SevScale for ARI patients across all ages reported to GIHSN. Upon implementation of the SevScale, contributing sites collecting data from hospitalized patients will be able to apply the GIHSN SevScale algorithm and upload virus sequence data to GISAID, accompanied by the corresponding patient Severity Score.

## 2 Methods

### 2.1 GIHSN Database

Data for this retrospective analysis were derived from the GIHSN database, which included prospectively collected information from influenza seasons starting in 2012/13. Over the years, the GIHSN grew to incorporate 24 participating sites worldwide, collecting case‐based data from eligible patients hospitalized with acute respiratory symptoms that could be associated with respiratory viruses, who were hospitalized for at least one night in any of the participating surveillance hospitals [10, 11]. Although specific enrolment strategies varied slightly per site and individual hospitals may have had different target populations, all participating sites shared a core protocol for enrolment of patients. Patients could be included in the study if they presented with community‐onset severe acute respiratory infection (SARI) based on predefined case definitions [14]. Samples were collected from patients who met the inclusion criteria and had given consent as approved by each participating site's Research Ethics Board. These samples were tested with real‐time reverse‐transcription polymerase chain reaction (RT‐PCR) for a selected number of respiratory viruses, varying by site. A standardized questionnaire was completed by trained healthcare workers at admission and again at death or hospital discharge to collect clinical outcomes. This questionnaire changed slightly over the years, and additional variables were added in later seasons. Collected data included symptoms at admission, patient characteristics, (pharmacological) treatment, influenza vaccination status, disease characteristics, and clinical outcome (Table S1).

### 2.2 Study Design and Population

For this retrospective observational pilot study, we included data from a geographically and economically diverse group of countries for the development of the SevScale, with countries from various WHO transmission zones [15] and income levels [13] and data for at least 2000 ARI patients over at least four seasons in the GIHSN database. Data from Brazil, Canada, Côte d'Ivoir, India, Kenia, Russia, South Africa, Spain, and Türkiye were included in the analysis. An anonymized dataset of all patients in the selected countries was drawn from the GIHSN database for this study. When a country had more than one participating site, data from those sites were combined to represent the country (Russia). Per their protocol, Brazil only registered pediatric patients (age < 18), while Canada registered exclusively patients 16 years or older. For Russia, only data collected before February 2022 were included.

### 2.3 Descriptive Analysis

We performed descriptive analyses regarding patient characteristics per country and availability of variables categorized as severity indicators in the GIHSN database, and chronic conditions using R version 4.1.2 [16]. The following variables were included in the analysis: country, season, influenza test result (sample collection date between 7 days before and 3 days after hospital admission), age, sex, pregnancy status (for women of reproductive age [17]), chronic conditions, and influenza vaccination status. Additionally, we evaluated data availability for the variables that were to be used as items in the model.

### 2.4 Model Development and Application

The SevScale model is a scale construction model, based on Item Response Theory (IRT) [18, 19, 20]. An IRT model uses items to indirectly measure a latent variable on a Logit scale, in this case severity. It has two levels: (1) the individual items and (2) overall random effects between patients, which are used to estimate the underlying Severity Score per individual. A general item response model was developed for the full dataset, using MLwiN software, with 15 indicators possibly associated with severity, from the database as items (confusion/lethargy, apnea (< 5 years old), vasopressor support, supplemental oxygen, frailty score (≥ 50 years old), oxygen saturation, blood pressure, respiratory rate, admission to high dependency unit, admission to intensive care unit, mechanical ventilation, length of hospital stay, discharge to another hospital, and death while hospitalized) (see Appendix S1 for model description). The Severity Score per individual is constructed based on the overall average severity (fixed effect) over all items for that individual. Negative estimates indicate that, compared to the average, this item is associated with increased severity. It should be noted that every item gets the same weight in contributing to the overall average and gets its own fixed effect item parameter (leaving out one item, the reference item) the so called “item difficulty parameter”. This is to control for the fact that items do not have the same probability of occurring. Next, the individual structural deviation from the overall average, the between individuals variation component in the model (random effect), is added. Continuous or categorical item variables with more than two categories were dichotomized for use in the model (e.g., blood pressure was divided into blood pressure < 130/80 and systolic blood pressure ≥ 130 OR diastolic blood pressure ≥ 80) based on literature [21, 22, 23, 24, 25]. We also determined the internal consistency of the model through the reliability coefficient, a measure for the performance of the scale in capturing the “true” differences between individuals on a scale between 0 and 1. A coefficient of 0.7 or higher indicates a statistically good measurement process [3].

Each ARI patient in the dataset received an individual Severity Score based on the items present for that patient and the overall average, quantifying the level of severity. This score represents a patient's place on the SevScale. The raw score was divided into a three‐category score to allow easier interpretation. Cutoff values were based on the percentile of patients in each category. For the purpose of this study, the 50% of patients with the lowest scores were placed in the Low Severity Category, the next 25% in the Medium Severity Category, and the 25% with the highest Severity Score in the High Severity Category. It is of note that all patients, including those in the Low Severity Category, can be considered severely ill compared to nonhospitalized patients.

### 2.5 Subgroup Comparison

In order to assess the distinguishing performance of the model, we compared the median Severity Score and the percentage of patients per Severity Category between different subgroups. Because the aim was to determine if the model was successful in showing differences between subgroups, rather than to interpret model outcomes, no multivariate analysis was performed. The subgroups in our study included influenza test result (positive, negative), influenza type (influenza A not subtyped, A(H1N1)pdm09, A(H3N2), B no lineage determined, B/Victoria, B/Yamagata), positive test for any respiratory virus (positive for at least one virus, negative for all) sex (male, female), age group (0–4, 5–17, 18–49, 50–64, 65+ years old), number of chronic conditions (none, one, two or more), pregnancy (pregnant, not pregnant), country income level (high income, upper‐middle income, lower‐middle income), and hospitalization pre‐ or since the COVID‐19 pandemic (admitted < 2020 W01, admitted ≥ 2020 W01).

## 3 Results

### 3.1 Descriptive Analysis

**FIGURE 1:**
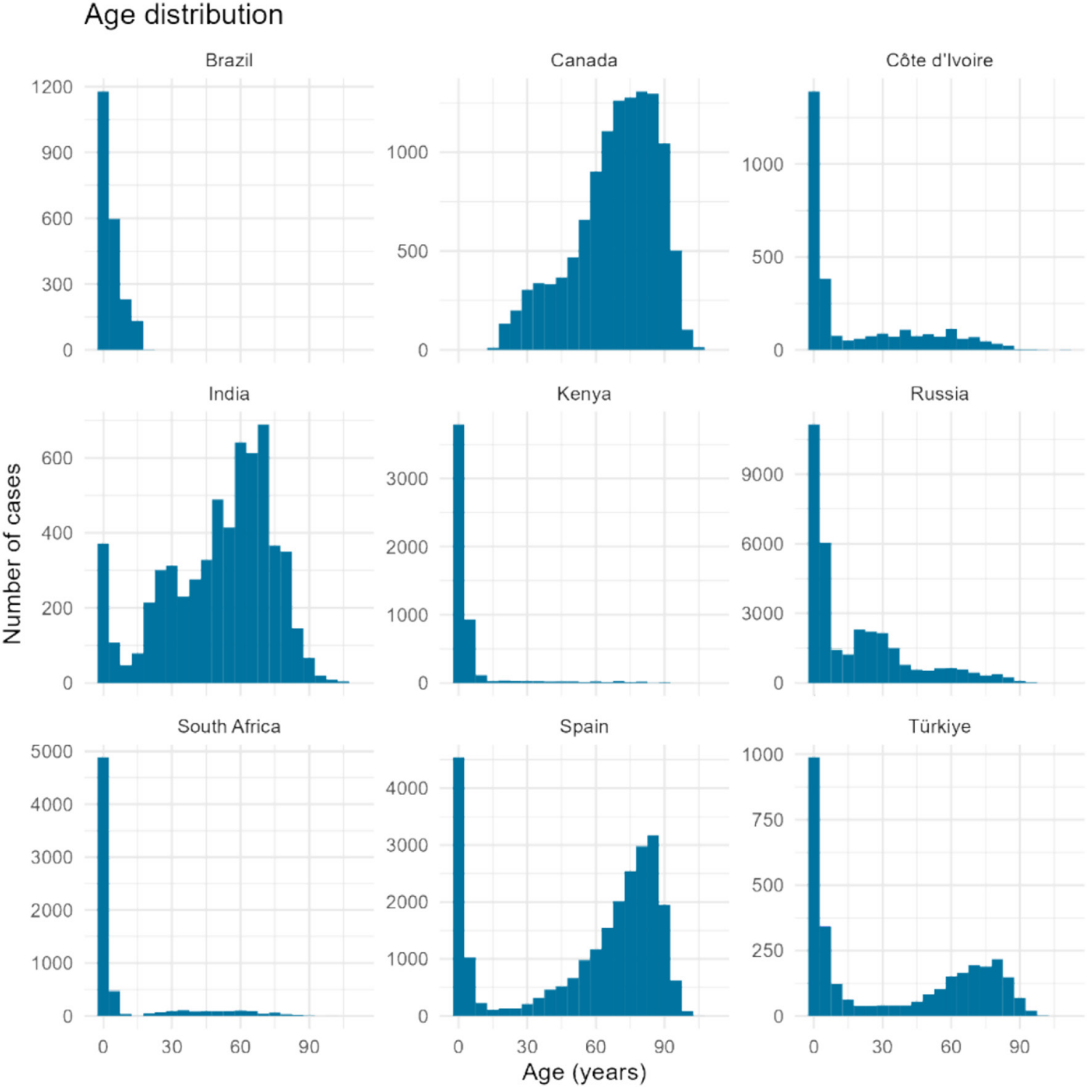
Age distribution of patients with acute respiratory infection per country.

Nine countries, from nine unique WHO transmission zones and three different income levels, were selected for the development of the GIHSN SevScale (Table 1), resulting in a total of 96,190 included patients. The overall median age of the population was 27 years (IQR 2–68), varying between < 1 (IQR: 0.6–5) in South Africa and 72 (IQR: 58–83) in Canada (Table 2). Age distribution of patients varied per country (Figure 1). Of patients with known influenza vaccination status, 17% had been vaccinated for the season of their hospital admission and at least 14 days before onset of respiratory symptoms. Among patients with known data for chronic condition status, 64% reported at least one chronic condition. Overall, 21% of the patients in the dataset tested positive for influenza, ranging from 5% (Côte d'Ivoire) to 31% (Russia).

**TABLE 1:** Countries and seasons included in the analysis.

Income group^a^	Country	WHO transmission zone	Number of seasons (seasons^b^)	*N* patients
High income	Spain	South West Europe	11 (2012/13–2022/23)	25,419
	Canada	North America	5 (2017/18–2021/22)	11,631
Upper‐middle income	Russia	Eastern Europe	9 (2013/14–2021/22)^c^	33,280
	Türkiye	Western Asia	8 (2013/14–2016/17 & 2019/20–2022/23)	3145
	Brazil	Tropical South America	4 (2018/19–2021/22)	2139
	South Africa	Southern Africa	5 (2016/17–2018/19 & 2020/21–2021/22)	6437
Lower‐middle income	Kenya	Eastern Africa	6 (2017/18–2022/23)	5169
	India	Southern Asia	8 (2015/16–2022/23)	6078
	Côte d'Ivoire	Western Africa	6 (2017/18–2022/23)	2892

^a^ Income group as defined by the World Bank, 2023.

^b^ For season 2022/23 data until week 3‐2023 are included.

^c^ For Russia, data until week 4‐2022 are included.

**TABLE 2:** Population characteristics per country.

Country	Median age (IQR)^a^	% female	Chronic condition^b^ *n/N* (%)	Pregnant^b^ *n/N* (%)	Vaccinated^b^, ^c^ *n/N* (%)
Brazil	2 (0.6–5)	48%	983/2028 (48)	0/408 (0)	742/2028 (37)
Canada	72 (58–83)	47%	8462/11,523 (73)	130/4962 (3)	3443/11,158 (31)
Côte d'Ivoire	3 (1–36)	46%	321/2346 (14)	3/815 (0)	40/2879 (1)
India	55 (35–70)	48%	4339/5595 (78)	99/2549 (4)	372/6078 (6)
Kenya	1.1 (0.5–2.8)	48%	1644/4721 (35)	33/1108 (3)	9/5169 (0)
Russia	6 (1.7–29)	50%	6059/16,720 (36)	3274/12,732 (26)	1335/33,151 (4)
South Africa	0.5 (0–2.2)	45%	1706/3598 (47)	37/1441 (3)	8/6435 (0)
Spain	69 (32–82)	46%	17,724/19,196 (92)	59/10,805 (1)	9967/25,386 (39)
Türkiye	22 (1–69)	45%	1912/2136 (90)	7/1397 (1)	320/3052 (10)
Total	27 (2–68)	48%	43,150/67,863 (64)	3642/36,217 (10)	16,236/95,336 (17)

^a^ In Brazil, only pediatric patients were included, with a maximum age of 18 years old. In Canada, only adult patients were included, with a minimum age of 16 years old.

^b^ Number of patients with chronic condition, pregnant patients, and patients vaccinated for influenza among those for which the variable was reported.

^c^ Vaccinated for the season of hospital admission.

### 3.2 Severity Indicators

Data availability for the severity indicators ranged widely between different variables (see Table 3). In general, the indicators measured during the hospitalization period or at discharge were reported more consistently than those measured at admission. Indicators with the highest data availability were intensive care unit (ICU) admission (97%), mechanical ventilation (88%), duration of hospital stay (95%), and death while hospitalized (97%).

**TABLE 3:** Presence and reporting completeness of severity indicators.

	Severity indicator	Reported *N*/total (%)	Prevalence *n/N* (%)
At admission	Confusion/lethargy	35,092/96,190 (36)	4758/35,092 (14)
	Apnea (< 5 years old)	9296/35,079 (27)	244/9296 (3)
	Vasopressor support	39,725/96,190 (41)	1558/29,725 (4)
	Supplemental oxygen	50,069/96,190 (52)	15,800/50,069 (32)
	Frailty score (50 + years old)	10,493/37,082 (28)	
	< 5		5063/10,493 (48)
	≥ 5		5430/10,493 (52)
	Oxygen saturation	30,471/96,190 (32)	
	< 90%		5274/30,471 (17)
	≥ 90%		25,197/30,471 (83)
	Blood pressure For age ≥ 13	29,167/96,190 (30) 20,133/55,637 (21)	
	< 130/80		19,330/29,167 (66) 11,198/20,133 (56)
	Systolic ≥ 130 and/or Diastolic ≥ 80		9837/29,167 (34) 8935/20,133 (44)
	Respiratory rate	35,476/96,190 (27)	
	Within normal range^a^		22,826/35,476 (64)
	Outside normal range		12,650/35,476 (36)
During hospital stay	High dependency unit	9951/96,190 (10)	5331/9951 (54)
	ICU admission	93,137/96,190 (97)	7206/93,137 (8)
	Mechanical ventilation	85,009/96,190 (88)	3195/85,009 (4)
Discharge	Length of stay	91,672/96,190 (95)	
	< 10 days		72,320/91,672 (79)
	≥ 10 days		19,352/91,672 (21)
	Frailty score (50 + years old)	4130/37,082 (11)	
	< 5		1609/4130 (39)
	≥ 5		2521/4130 (61)
	Discharged to another hospital	38,165/96,190 (40)	1067/38,165 (3)
	Death while hospitalized	93,603/96,190 (97)	3935/93,603 (4)

^a^ See Table S2 for age‐dependent normal range.

Abbreviation: ICU, intensive care unit.

### 3.3 Model Results and Scale Development

The model estimates for the severity indicators are described in Table S3. Items such as “Died in hospital” and “Mechanical ventilation” had a lower estimate and are thus associated with higher severity than “high blood pressure,” “abnormal respiration rate,” “oxygen support,” and “frailty score ≥ 5.” The current model has a reliability coefficient of 0.639.

**FIGURE 2:**
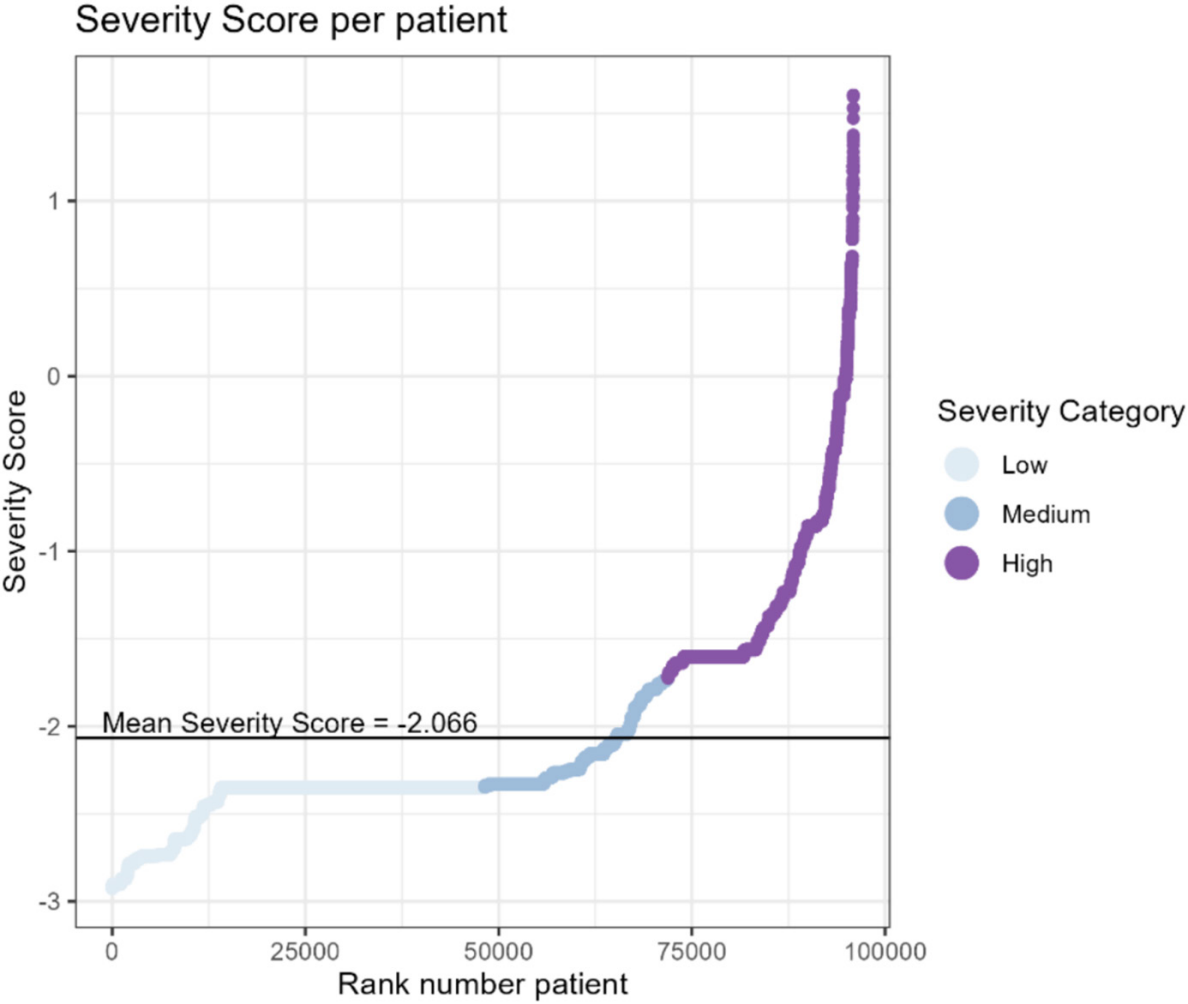
Severity Score distribution. Displaying patients ranked from lowest to highest Severity Score (*x*‐axis), with their corresponding Severity Score.

The Severity Score estimates for the patients in the dataset showed variability, ranging between −2.92 and 1.60 with a mean score of −2.07 (Figure 2). This is a mathematically constructed score on a logit scale, and therefore, an individual's score itself yields little interpretable information. When comparing scores, a higher score indicates higher severity. We categorized 50% of patients (*n* = 48,210) in the Low Severity Category 1 (≤ − 2.351), 25% (*n* = 23,713) in the Medium Severity Category 2 (> − 2.351 and ≤ − 1.726), and 25% (n = 23,969) High Severity Category 3 (> − 1.726). The widest range of Severity Scores can be found in Severity Category 3 (−1.726–1.60).

### 3.4 Behavior of Model Score in Different Subgroups

Patient groups with a higher percentage of patients in the High Severity category were those positive for influenza A(H1N1)pdm09 (compared to others with known influenza subtype or lineage), those vaccinated for influenza, those aged 50 years or older, those from lower‐middle income countries, and those admitted since the start of the COVID‐19 pandemic (Table 4). The percentage of patients in the High Severity Category increased with the number of chronic conditions (none, one, or more than one chronic condition). Patient groups with more patients in the Low Severity category were those with a positive influenza test, those tested positive for any virus, and pregnant patients.

**TABLE 4:** Percentage of patients per severity category, per subgroup.

	*N* patients	Severity category		
		Low	Medium	High
Flu positive				
Yes	19,734	12,297 (62.3%)	4008 (20.3%)	3429 (17.4%)
No	33,173	12,625 (38.1%)	10,828 (32.6%)	9720 (29.3%)
Influenza type				
A not subtyped	1756	912 (51.9%)	337 (19.2%)	507 (28.9%)
A(H1N1)pdm09	4802	2925 (60.9%)	1058 (22.0%)	819 (17.1%)
A(H3N2)	5744	3777 (65.8%)	1073 (18.7%)	894 (15.6%)
B lineage not determined	1431	1011 (70.6%)	214 (15.0%)	206 (14.4%)
B/Victoria	1474	1067 (72.4%)	207 (14.0%)	200 (13.6%)
B/Yamagata	1631	1162 (71.2%)	222 (13.6%)	247 (15.1%)
Positive for any respiratory virus				
Yes	45,623	22,738 (49.8%)	11,156 (24.5%)	11,729 (25.7%)
No	271	72 (26.6%)	103 (38.0%)	96 (35.4%)
Vaccinated for influenza				
Yes	12,236	7899 (48.7%)	3187 (19.6%)	5144 (31.7%)
No	73,592	38,800 (52.7%)	18,182 (24.7%)	16,610 (22.6%)
Sex				
Female	46,005	24,879 (49.9%)	12,205 (24.5%)	12,794 (25.7%)
Male	49,878	23,331 (50.7%)	11,503 (25.0%)	11,171 (24.3%)
Age group				
00–04	35,003	19,213 (54.9%)	9863 (28.2%)	5927 (16.9%)
05–17	7005	4196 (59.9%)	1728 (24.7%)	1081 (15.4%)
18–49	16,820	9569 (56.9%)	4037 (24.0%)	3214 (19.1%)
50–64	9772	3874 (39.6%)	2340 (23.9%)	3558 (36.4%)
65+	27,278	11,353 (41.6%)	5743 (21.1%)	10,182 (37.3%)
Chronic conditions				
None	53,252	30,705 (57.7%)	14,447 (27.1%)	8100 (15.2%)
One	19,123	8641 (45.2%)	4786 (25.0%)	5696 (29.8%)
Two or more	23,517	8864 (37.7%)	4480 (19.1%)	10,173 (43.3%)
Pregnant				
No	23,091	12,126 (52.5%)	5221 (22.6%)	5744 (24.9%)
Yes	3642	2600 (71.4%)	804 (22.1%)	238 (6.5%)
Income level country				
High income	37,027	17,723 (47.9%)	8357 (22.6%)	10,947 (29.6%)
Upper‐middle income	44,800	26,492 (59.1%)	10,906 (24.3%)	7402 (16.5%)
Lower‐middle income	14,065	3995 (28.4%)	4450 (31.6%)	5620 (40.0%)
COVID‐19 pandemic				
Prepandemic	59,970	36,340 (60.6%)	11,211 (18,7%)	12,419 (20.7%)
Since start pandemic	35,715	11,868 (33.2%)	12,321 (34.5%)	11,526 (32.3%)
Total	95,892	48,210 (50.3%)	23,713 (24.7%)	23,969 (25.0%)

## 4 Discussion

The GIHSN SevScale model was developed for ARI patients of all ages, using patient data from the full hospitalization period to construct a Severity Score. The SevScale included 15 items that are readily collected during a hospitalization and stratified patients into three severity categories. The model also highlighted disparities in severity across patient subgroups and country characteristics, such as those with different influenza subtypes or lineages, number of chronic conditions, or country income level.

When comparing subgroups, the model showed variation in the proportion of patients categorized in the Low, Medium, and High Severity Categories. In this study, the allocation of patients to each category was arbitrarily based on the distribution of Severity Scores. Although no multivariate analysis was performed, robustness of data is inferred from the fact that these differences are in the expected direction, they are clinically‐plausible, and in line with previous publications: The subgroups studied by Cohen et al. had outcomes comparable to ours [26]. For example the finding that influenza A(H1N1)pdm09 was related to higher severity than A(H3N2), increased mortality for older patients and those with underlying chronic conditions, and increased risk of ICU admission in LMIC [26]. Although unexpected, the finding that a higher percentage of people vaccinated for influenza is in the High Severity Category is in accordance with a number of previous findings for nonhospitalized ARI [27]. Nonetheless, this finding might well be a result of confounding as no multivariate analysis was performed and influenza vaccine coverage was higher in the 65 + age group and people with chronic conditions. In addition, pregnancy is known to be associated with an increased risk of hospitalization and mortality due to influenza [28, 29]. The lower percentage of pregnant patients in the High Severity Category could be a result of a more lenient admission policy for pregnant patients with ARI.

The low percentage of data availability in some severity indicators can largely be explained by changes in the data collection protocol. Many variables with low data availability in our study started being collected in more recent seasons (2018/19 and onwards). Moreover, data reporting policies varied in the participating sites and not all sites collected all variables. For example, a high dependency unit does not exist in all hospitals; possibly, this variable is left empty in the reporting stage when not applicable. Similarly, data for certain chronic conditions were not collected until the 2018/19 season or later. Additionally, variables related to chronic conditions may mistakenly be left empty at the reporting stage when not applicable. Information on chronic conditions is vital for the model's future adjustment. Ongoing improvements in data quality and completeness of patients' clinical outcomes will help increase the accuracy of the SevScale model.

The SevScale algorithm produced a retrospectively constructed Severity Score that can be used in various settings. Primarily, it can become an integrated part of the GIHSN database and potentially other surveillance networks. The score will be calculated using the model estimate based on the existing patients in the dataset. To calculate the Severity Score, a new patient should have a valid answer for at least one of the 15 items. The score will be calculated using the estimate from the model, based on the existing patients in the dataset. Thus, the SevScale can add another layer of information to surveillance efforts and aid in decision making regarding influenza vaccine strain selection. The Severity Score and viral genetic sequencing data can easily be linked by adding the Severity Score information to the viral sequences shared with GISAID. The availability of the additional information provided by the Severity Score in GISAID will allow the WHO Collaborating Centers (and other research groups working with the GISAID database) to identify the main characteristics of the severe influenza patients and, consequently, to take patient severity into account for viral monitoring and for selecting strains for the influenza vaccine. The difference in Severity Score between influenza types, with influenza A(H1N1)pdm09 associated with higher severity, indicates relevant variation between influenza subtypes, which potentially can also be found between strains.

The Severity Score could also be utilized as an outcome variable in research efforts, studying severity of ARI or specific respiratory viruses. This would provide a more comprehensive definition of severity than a clinical score or single proxy for severity and enhance the comparability of different studies analyzing the severity of influenza or other ARIs. Another potential application could be to compare the distribution of ARIs in different hospitals according to the Severity Score or compare the severity of influenza in vaccinated and unvaccinated patients. When linked to relevant clinical outcomes, the Severity Score could also be used in assessments of the cost‐effectiveness of hospitalization of ARI patients.

The development of this model stands out from other studies of ARI or influenza disease severity, as it does not aim to identify predictors of severity but rather to quantify the concept of severity itself. The GIHSN SevScale is explicitly not suitable as a SevScale for the clinical management of patients as the model is based on data from the entire hospitalization period, and the score is assigned retrospectively. Clinical management scales, such as the CURB‐65 for community‐acquired pneumonia and others used in ARI study settings, already exist and serve a different purpose than the GIHSN SevScale [30]. To our knowledge, the only other SevScale for the same purpose is the ViVI score developed by Rath et al. in 2017, using data collected at emergency department visit or pediatric inpatient ward for a composite score [12]. Unlike the scale developed by Rath et al., the SevScale does not require information on all severity indicators, although reliability increases with more information.

The most important limitation affecting the development of the SevScale is the lower‐than‐expected data availability. This decreases the model's reliability because the number of patients with each indicator is suboptimal. Moreover, further specification of the SevScale was impossible, as the number of occurrences of each severity indicator would be too low to yield a reliable influenza‐ or age‐specific model. Secondly, the inherent heterogeneity of the data over the various countries and seasons impacts the model's reliability and therefore increases necessity for a high number of patients to develop the model. Thirdly, no low income countries could be included in the study, which reduces the generalizability of the SevScale to these economies. Another limitation is the fact that the SevScale was developed using a set of currently available parameters from clinical practice. These parameters may change in the future, or new and better ways to measure severity may be developed or more widely used. In that case, the current SevScale would be no longer sufficient and would have to be updated.

The next step in the development and implementation of the SevScale should be to recalibrate and validate the model, applying it to the full GIHSN database and adding more recent data. More complete and representative data are expected to increase the model's reliability and allow further specifications and adjustments according to patient characteristics. Since there is no gold standard for disease severity in hospitalized ARI patients, the validation aims to establish if the model results comply with expectations based on current knowledge. This is a vital step in determining whether the model correctly measures the concept of severity. A proposed number and allocation of Severity Categories could be included in this validation step or determined by researchers based on clinical significance.

Multiple years of data are necessary for the model development to include the required number of patients and to account for between‐season variation. Over time, GIHSN data collection became more extensive, and we expect data availability to be higher when more recent seasons are selected. The addition of new data to the database and continued improvements would allow the SevScale model to be “finetuned.” A follow‐up study would also offer the opportunity to explore if a minimum subset of items should be determined. Since item estimates can be influenced by age, country, and comorbidities, adjusting the model for these factors should be possible. To calculate this adjusted score, a regression model can be developed with the Severity Score from the database and the extra variables (e.g., age groups and chronic condition) to produce parameters that capture this variation in severity.

In conclusion, the pilot model was successful in proving the concept of quantifying severity in hospitalized patients with ARI for surveillance purpose. Repeating this exercise with the full database and adding new, more complete data will be necessary to allow recalibration and validation of the current model. Nevertheless, this model proved to be a promising method to define severity for influenza vaccine strain selection, surveillance and research.

## Author Contributions

**Bronke Boudewijns:** data curation, formal analysis, visualization, writing – original draft, writing – review and editing. **Saverio Caini:** conceptualization, supervision, writing – review and editing. **Marco Del Riccio:** conceptualization, writing – review and editing, supervision. **Marta C. Nunes:** writing – review and editing. **Sandra S. Chaves:** writing – review and editing. **Melissa K. Andrew:** writing – review and editing. **Justin R. Ortiz:** writing – review and editing. **Oana Săndulescu:** writing – review and editing. **Joseph S. Bresee:** writing – review and editing. **Elena Burtseva:** writing – review and editing, data curation. **Daouda Coulibaly:** data curation, writing – review and editing. **Daria M. Danilenko:** data curation, writing – review and editing. **Kirill Stolyarov:** data curation, writing – review and editing. **Anca C. Drăgănescu:** writing – review and editing. **Mine Durusu Tanriover:** data curation, writing – review and editing. **Heloisa I. G. Giamberardino:** data curation, writing – review and editing. **Parvaiz A. Koul:** data curation, writing – review and editing. **F. Xavier Lopez‐Labrador:** data curation, writing – review and editing. **Shelly A. McNeil:** data curation, writing – review and editing. **Ainara Mira‐Iglesias:** data curation, writing – review and editing. **Alejandro Orrico‐Sanchez:** data curation, writing – review and editing. **Nancy A. Otieno:** data curation, writing – review and editing. **Jorim Ayugi:** data curation, writing – review and editing. **Sonia M. Raboni:** data curation, writing – review and editing. **Peter Spreeuwenberg:** conceptualization, formal analysis, writing – review and editing, methodology.

## Ethics Statement

The surveillance protocol used by the GIHSN was approved by each participating site's Research Ethics Board.

## Consent

Informed consent was obtained from all patients and/or their legal guardian(s) included in the study.

## Conflicts of Interest

Sandra S. Chaves is an employee of Sanofi, but the content of this paper is not representative of the views of their organization. Sandra S. Chaves contributed to the interpretation of results and the writing of the manuscript and report as a member of the Foundation for Influenza Epidemiology. Oana Săndulescu and Anca C. Drăgănescu report being investigators in the DRIVE project that has received support from the EU/EFPIA Innovative Medicines Initiative 2 Joint Undertaking (grant no. 777363) outside the scope of the submitted work and the GIHSN project, which was cofunded by the Foundation for Influenza Epidemiology, France, within the scope of the submitted work. Melissa K. Andrew and Sonia M. Raboni report grants from GSK, Sanofi, Pfizer, and Merck, unrelated to the present study. Marta C. Nunes reports grants from the Sanofi, unrelated to the present study, and personal fees from Pfizer and Sanofi, unrelated to the present study. F. Xavier Lopez‐Labrador reports grants and personal fees from Sanofi, unrelated to the present study. Shelly A. McNeil reports grants from Merck, Pfizer, and GSK unrelated to the present study and personal fees from Pfizer, Moderna, Merck, Sanofi, and GSK, unrelated to the present study. Bronke Boudewijns, Saverio Caini, Marco Del Riccio, Justin R. Ortiz, Joseph S. Bresee, Elena Burtseva, Daouda Coulibaly, Daria M. Danilenko, Kirill Stolyarov, Mine Durusu Tanriover, Heloisa I. G. Giamberardino, Parvaiz A. Koul, Ainara Mira‐Iglesias, Alejandro Orrico‐Sanchez, Nancy A. Otieno, Jorim Ayugi, and Peter Spreeuwenberg report no conflict of interest.

### Peer Review

The peer review history for this article is available at https://www.webofscience.com/api/gateway/wos/peer‐review/10.1111/irv.70085.

## Supporting information


**Appendix S1** Item Response Model.
Appendix S2 supplementary tables
Table S1 Questions from the GIHSN questionnaire1.1 The GIHSN SevScale focuses on the shaded questions
Table S2 respiratory rate reference ranges.
**Table S3.** SevScale model results.

## Data Availability

Anonymized data used for this analysis, along with a data dictionary, are available on request to contact@gihsn.org. The use of data depends on the approval of an analytical proposal by the Independent Scientific Committee. Investigators from participant sites are informed up front for any planned data analysis, and they have the possibility to opt out.
