# Supplementary material for: Severity Scale of Influenza and Acute Respiratory Illness Hospitalizations to Support Viral Genomic Surveillance: A Global Influenza Hospital Surveillance Network Pilot Study
Source: Influenza Other Respir Viruses. 2025 Mar 6;19(3):e70085. doi: 10.1111/irv.70085 (PMC11883289; doi:10.1111/irv.70085)
Supplement: Supplementary file 1 — Appendix S1 Item Response Model. Appendix S2 supplementary tables Table S1 Questions from the GIHSN questionnaire1.1 The GIHSN SevScale focuses on the shaded questions Table S2 respiratory rate reference ranges. Table S3. SevScale model results. [file IRV-19-e70085-s001.docx]

**Appendix 1; Item Response Model**

Statistical model: IRT multilevel logistic model

Y_h,i_ = β_0_+β_(h 2..15)_item(h,i)+µ_(i)_+є

Y_h,i_ : score on an item for every individual.

h: items

i: individuals

β_0_ : intercept on the scale

β_(h 2..15)_item(h,i) : regression coefficients for the items 2-15. Item 1 is the reference item and therefore left out. These item parameters represents what in IRT models is often called the item difficulty parameter. The 0/1 item indicators are rescaled by subtracting 1/15.

µ_(i)_ : between individual variance

є : error variance, for a logistic model (binomial distribution, parameter constrained to 1, with a logit link function, estimation procedure: RIGLS , PQL second order, analyzed with MLwiN software)

Scale score per individual is the summation of two model components:

Scale score per individual = β_0_+µ_(i)_

**Appendix 2; supplementary tables**

*Supplementary table S1 Questions from the GIHSN questionnaire^1^*

| **Questionnaire item** | **≥5 years of age** | **Children <5** |
| --- | --- | --- |
| **I. Admission characteristics:** |  |  |
| Admission code / ICD | √ | √ |
| Age | √ | √ |
| Sex | √ | √ |
| Influenza virus type/subtype/B lineage | √ | √ |
| Co-infection (8 other viruses and other) | √ | √ |
|  |  |  |
| **II. Patient characteristics:** |  |  |
| Height/weight/BMI | √ | √ |
| Chronic condition (various) | √ | √ |
| Pregnancy | √ |  |
| Vaccination status | √ | √ |
|  |  |  |
| **III. Treatment:** |  |  |
| Antivirals | √ | √ |
| Antibiotics | √ | √ |
|  |  |  |
| **IV. Severity:** |  |  |
| Confusion/lethargy at admission | √ | √ |
| Oxygen saturation value on ambient air (%) | √ | √ |
| Blood pressure | √ | √ |
| Supplemental oxygen without mechanical ventilation | √ | √ |
| Vasopressor support | √ | √ |
| Apnea (measured at admission) |  | √ |
| Respiratory rate (measured at admission) | √ | √ |
| ICU admission (measured at any time) | √ | √ |
| Mechanical ventilation (measure at any time) | √ | √ |
| Baseline frailty score at admission (patients 50 and older) | √ |  |
|  |  |  |
| **V. Discharge:** |  |  |
| Diagnosis at discharge (Main/Secondary 1/Secondary 2) | √ | √ |
| Length of stay (days) | √ | √ |
| Discharge to another hospital | √ | √ |
| Death while hospitalized | √ | √ |
| Frailty score at discharge (patients 50 and older) | √ |  |

^1^ The GIHSN SevScale focuses on the shaded questions

*Supplementary table S2 respiratory rate reference ranges*

| Respiratory rate | |
| --- | --- |
| **Age (years)** | **Normal range (breaths per minute)** |
| 0-2 | 20-40 |
| 2-5 | 20-30 |
| 6-9 | 18-25 |
| 10-17 | 17-23 |
| 18-49 | 15-18 |
| 50-64 | 18-25 |
| 65-79 | 12-28 |
| 80+ | 10-30 |

*Supplementary table S3 SevScale model results*

| **Item** | **N patients** | **% of total patients** | | **Estimate** | **Standard error** |
| --- | --- | --- | --- | --- | --- |
|  |  | | Random part | | |
| Between patient variance |  |  | | 0.984 | 0.012 |
| Error |  |  | | 1 | 0 |
|  |  | | Fixed part | | |
| Intercept |  |  | | -2.066 | 0.008 |
| Length of stay | 93554 | 97.3 | |  |  |
| Oxygen saturation | 30471 | 31.7 | | -0.447 | 0.018 |
| Blood pressure | 15332 | 15.9 | | -0.066 | 0.022 |
| Respiratory rate | 35468 | 36.9 | | 0.493 | 0.014 |
| Supplemental oxygen | 39534 | 41.1 | | 0.710 | 0.013 |
| Vasopressor support | 39726 | 41.3 | | -2.074 | 0.029 |
| ICU admission | 93138 | 96.8 | | -1.243 | 0.015 |
| Death while hospitalised | 93604 | 97.3 | | -1.891 | 0.018 |
| Mechanical ventilation | 85010 | 88.4 | | -2.017 | 0.020 |
| Frailty at admission (≥ 50) | 15335 | 41.4 | | 0.548 | 0.020 |
| Confusion/lethargy | 41304 | 42.9 | | -0.923 | 0.018 |
| Apnea (<5) | 13923 | 39.7 | | -2.608 | 0.060 |
| Discharged to another hospital | 31771 | 33.0 | | -2.283 | 0.035 |
| Frailty at discharge (≥ 50) | 7609 | 20.5 | | 0.625 | 0.026 |
| High dependency unit | 5112 | 5.3 | | -1.251 | 0.054 |
